# Development of chicken tender pops by utilizing pomegranate peel powder

**DOI:** 10.1002/fsn3.3412

**Published:** 2023-05-22

**Authors:** Zunaira Basharat, Maryam Imran, Naeem Fatima, Muhammad Wasim Sajid, Muhammad Rizwan Tariq, Shinawar Waseem Ali, Zujaja Umer, Waseem Safdar, Humphrey Garti

**Affiliations:** ^1^ Department of Food Sciences University of the Punjab, Quid‐i‐Azam Campus Lahore Pakistan; ^2^ Sharif Medical and Dental College Lahore Lahore Pakistan; ^3^ Allied Hospital Faisalabad Faisalabad Pakistan; ^4^ Department of Biosciences COMSATS University Islamabad, Sahiwal Campus Sahiwal Pakistan; ^5^ Department of Biological Sciences National University of Medical Sciences Rawalpindi Pakistan; ^6^ Department of Nutritional Sciences University for Development Studies Tamale Ghana

**Keywords:** chicken tender pops, functional food, microbiological evaluation, oxidative potential, pomegranate Peel powder, sensory profile, storage stability

## Abstract

Pomegranate peel powder (PPP) is a rich source of many bioactive components particularly polyphenols that are interlinked to various technological and functional properties. In the present study, chicken tender pops were developed with incorporation of PPP, and its effect on quality attributes and storage stability of the product were evaluated. The treatments were formulated using 0%, 3%, 6%, and 9% PPP in replacement of chicken. The physicochemical properties, texture profile, instrumental color, sensory attributes, and storage stability were assessed for 21 days at refrigeration temperature, at a regular interval of 7 days. The results indicated that the inclusion of PPP significantly (*p* < .05) increased the dietary fiber from 0.25% in T_0_ to 1.45% in T_3_ at Day 0 and WHC 43.60% ± 0.02 in T_0_ to 49.36% ± 0.02 in T_3_ at Day 0, whereas the moisture content significantly reduced from 60.05% ± 0.03 in T_0_ to 55.08% ± 0.01 in T_3_ at the start of the study. In addition, the values of TBARS were significantly (*p* < .05) reduced for treated samples 0.72 mg MDA/Kg in T_3_ as compared to control 1.17 mg MDA/Kg on the 21st day of storage, whereas a significant increase (*p* < .05) in TPC from 0.90 mg GAE/g to 3.87 mg GAE/g in T_0_ to T_3_ was observed at the start of the study. For TPA, a significant (*p* < .05) increase was noticed in hardness, chewiness, and gumminess, whereas cohesiveness and springiness showed a non‐significant (*p* > .05) change in treated samples in relation to control, and the instrumental color (L* and a*) decreased significantly. However, pH, crude fiber, fat, ash, and protein content showed non‐significant (*p* > .05) variations over time. The sensory evaluation suggested that chicken tender pops supplemented with 6% PPP (T_2_) presented high overall acceptability and balanced organoleptic properties. Hence, it can be concluded that PPP can be effectively utilized as a natural fiber source, antioxidant, and antimicrobial agent in novel functional foods.

## Graphical abstract

The study evaluated the effects of incorporation of pomegranate peel powder on physiochemical, biochemical, and sensory attributes in development of chicken tender pops in refrigeration storage. Results show that use of pomegranate peel power enhanced antioxidant concentration, antimicrobial properties, sensory characteristics, and improved nutritional profiles while exhibiting potential of natural preservatives.

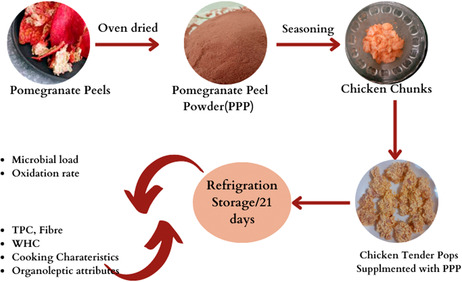

## 1 INTRODUCTION

Pomegranate (*Punica granatum*) is a spherical fruit that is a member of the Punicaceae family which was first cultivated in Iran and India and then spread throughout the Mediterranean basin (El Barnossi et al., 2021). Global pomegranate production is around 8.1 million tons, with a planting area of 835,950 hectares (Pienaar & Barends‐Jones, 2021). The global pomegranate market is anticipated to grow from 208.9 million USD in 2020 to 322.9 million USD by 2026 (River Country, 2021). Pomegranate fruit comprises two parts, an edible part that is 50% of the fruit, and the other 50% is the peel (Rafraf et al., 2017). Pomegranate is a valuable fruit because of its nutritional components such as minerals, proteins, crude fibers, vitamins, alkaloids, organic acids, fatty acids, flavonoids, polyphenols, isoflavones, and pectin that are associated with various therapeutic and technological benefits (Pirzadeh et al., 2021; Rahmani et al., 2017; Viuda‐Martos et al., 2010). It is usually consumed fresh or processed into different products such as juice, jam, oil, wine, or dietary supplements (Kahramanoglu & Usanmaz, 2016). However, the industrial processing of pomegranate produces enormous amounts of by‐products, mainly peels (40%–50%) which are disposed of as waste without any valorization that jeopardizes the environment (Ali et al., 2019). On the other hand, Pomegranate peel can be valorized to produce pomegranate peel powder and peel extracts containing many functional biomolecules (Jalal et al., 2018; Singh et al., 2019). These valorized products could be incorporated into the food chain to promote bio‐economy and satisfy sustainable development principles (Ben‐Othman et al., 2020; Sharayei et al., 2019).

Poultry consumption, particularly chicken meat, is associated with many positive health benefits and is considered more valuable than other meats because of its low energy value with high nutritional density (Bordoni & Danesi, 2017; Millen et al., 2014). In addition, it contains considerable amounts of long‐chain n‐3 polyunsaturated fatty acids, trace minerals (Fe and Zn), and B group vitamins, along with minute amounts of biotin, folic acid, pantothenic acid, and vitamin E (Barroeta, 2007). However, chicken meat lacks the dietary fiber essential to maintain human health by avoiding various ailments (Verma & Banerjee, 2010). Furthermore, using artificial preservatives to preserve the nutritional and quality attributes of meat products is associated with negative health effects such as allergy, asthma, cancer, hyperactivity, hypersensitivity, and neurological damage (Anand & Sati, 2013; Smaoui et al., 2019). Therefore, meat industries and researchers are focused on discovering natural substitutes to replace these synthetic additives with renewable biomass that is a natural safe source of many functional biomolecules (Pateiro et al., 2018; Žugčić et al., 2019).

Pomegranate peels are an excellent source of minerals (calcium, magnesium, potassium, phosphorus, and sodium) (Jalal et al., 2018), bioactive peptides (Hernández‐Corroto et al., 2020), and polysaccharides (Zhu et al., 2015). Furthermore, it contains high volumes of phytochemicals, mainly flavonoids (anthocyanins, catechin, epicatechin, and gallocatechin), hydrolyzable tannins (ellagitannins and gallotannins), and phenolic acids (caffeic acid, ellagic acid, and gallic acid) (Kaderides et al., 2020). The presence of these bioactive compounds is associated with a diverse range of biological activities (antibacterial, antifungal, and antimicrobial), therapeutic properties (anticarcinogenic, antihypertensive, anti‐inflammatory, antimutagenic, and antioxidant), and technological functions in foods (antioxidant, antimicrobial, emulsifying agent, oil‐holding and water‐holding agent colorant, flavoring, and nutraceuticals). Furthermore, secondary metabolites promote the avoidance and treatment of many chronic conditions, such as Alzheimer's disease, cardiovascular diseases, diabetes, and obesity (Jalal et al., 2018; Kandylis & Kokkinomagoulos, 2020; Ko et al., 2021).

The current study is aimed to develop a functional meat product (chicken tender pops) by effectively utilizing pomegranate peel powder and exploring its efficacy as a natural dietary fiber source, antioxidant, and antimicrobial agent. Furthermore, the effect of PPP incorporation on physiochemical characteristics, proximate composition, cooking characteristics, oxidative stability, instrumental color, texture profile, and sensory attributes of chicken tender pops was also assessed to predict the product's storage quality at refrigeration for 21 days.

## 2 MATERIALS AND METHODS

### 2.1 Procurement of raw materials

The pomegranate (*Punica granatum*) was procured from the local market of Lahore, Pakistan. The fresh boneless broiler chicken meat was purchased from the local Superstore in Lahore, Pakistan. The meat was packed in small bags of LDPE and held in a refrigerator at 4 ± 2°C for 6 h and later used to develop chicken tender pops.

### 2.2 Preparation of pomegranate peel powder (PPP)

The PPP was prepared using the method of Jalal et al. (2018), with few modifications. Briefly, fresh pomegranates were washed thoroughly with distilled water to remove surface dust. The arils were separated from rinds and then cut into medium‐sized pieces. Pomegranate peels (rind) were placed in a tray and dried using a hot‐air oven DOF‐230E (Bievopeak, Japan) at 50 ± 2°C for 48 hrs. Dried peels were cooled and ground enough to pass through a 20‐mesh sieve to obtain a fine powder with uniform particle size. Pomegranate peel powder was transferred in zipped‐lock high‐density polyethylene bags and stored at room temperature 20 ± 3°C for physicochemical analysis and later used in product development.

### 2.3 Physicochemical analysis of pomegranate peel powder (PPP)

Proximate analysis (moisture, fat, protein, ash, and crude fiber) was performed using standard protocols of AOAC (2005). The pH of PPP was determined using a pH meter described by Jalal et al. (2018). Two gram of the sample was combined with 20 mL of methanol and left for 2 days to allow for maximum leaching to analyze the TPC of PPP. Folin–Ciocalteu reagent 1.5 mL was added into extract 0.5 mL and incubated at 25°C for 5 min. After incubation, 6% sodium carbonate 1.5 mL was added and incubated again for 90 min in a dark room. The absorbance of the resulting blue color mixture was measured at 725 nm, and the total phenolic content was articulated as mg GAE per 100 mL of a sample (Mahmoud et al., 2017).

### 2.4 Manufacturing of chicken tender pops

Treatments of chicken tender pops were prepared using various concentrations of PPP replacing chicken meat. 0%, 3%, 6%, and 9% PPP were added to 80%, 77%, 74%, and 71% boneless meat, respectively, while other ingredients were added according to weight ratio (w/w) mentioned in Table 1. Boneless chicken breasts were washed with tap water, dried using a paper towel, and sliced into chunks of 8 g to 10 g. Chicken chunks were uniformly mixed with PPP, onion powder, garlic powder, black pepper, paprika, and iodized salt with the addition of chilled water according to the formulation mentioned in Table 1. After a marinating stay of 30 min, chunks were coated with flour and coarsely ground cornflakes. The functional chicken tender pops were packaged aerobically in low‐density polyethylene (LDPE) boxes and stored in the refrigerator for 21 days. Physiochemical properties, proximate composition, cooking characteristics, oxidative stability, microbiological studies, texture profile, instrumental color, and sensory attributes of chicken tender pops were evaluated every 7 days up to 21 days. To determine sensory characteristics and cooking characteristics, the developed product was air fried using air fryer DWAF, 3013 (Dawlance, Pakistan) for 10 min at 200 ± 2°C.

**TABLE 1:** Formulation of chicken tender pops.

Ingredients	Treatments			
	T_0_	T_1_	T_2_	T_3_
Chicken breast (g)	80	77	74	71
Pomegranate peel powder (g)	0	3	6	9
Paprika (g)	2	2	2	2
Garlic powder (g)	2	2	2	2
Onion powder (g)	2	2	2	2
Black pepper (g)	2	2	2	2
Salt (g)	2	2	2	2
Chilled water (mL)	10	10	10	10

### 2.5 Determination of physiochemical parameters of chicken tender pops

The pH of chicken tender pops was estimated by the dipping probe of a digital pH meter (HANNA‐instrument, USA) in a homogenized sample by following the method of Santhi et al. (2020). The water‐holding capacity of samples was assessed as described by Rupasinghe et al. (2022), samples were placed between layers of filter paper and subjected to 10 kg weight for 5 min, and weight difference was expressed as WHC of samples.

### 2.6 Determination of proximate composition

Moisture, fat, protein, ash, and crude fiber content of chicken tender pops were determined by following the standard protocols of AOAC, 2005. Chopped samples of chicken tender pops were oven dried at 100°C for 2 h, cooled in a desiccator, and moisture content was measured as weight loss. The solvent extraction method was used to determine fat content; methanol was used as a solvent. Protein was assessed through the Kjeldahl method of digestion. Samples were subjected to a muffle furnace at a temperature of 550°C for ashing. For crude fiber determination, defatted samples were digested using acid and base. After digestion, the leftover material was weighed and ashed. The crude fiber was calculated as the difference in sample weight.

### 2.7 Determination of TPC and antioxidant activity (TBARS)

Folin–Ciocalteu method, as described by Firuzi et al. (2019), was used to determine the total phenolic content of the product with few modifications. 0.5 mL methanolic extract of the sample was mixed with 1.5 mL of Folin–Ciocalteu reagent and incubated at 25°C for 5 min. Afterward, 1.5 mL of sodium carbonate (6%) was added, and the sample was incubated in a dark room for about 90 min. The absorbance of the blue color mixture was taken at 725 nm, and total phenolic content was expressed as milligram gallic acid equivalents (GAE) per 100 mL of a sample. The assessment of TBARS was done to predict the lipid oxidation of the product. TBARS were measured using the method of Mashau et al. (2021) with few modifications, methanolic extracts of chicken tender pop samples were mixed with thiobarbituric acid, and centrifuged at 3000*g* for 15 min. Samples were heated at 95°C for 60 min in a water bath, followed by cooling at 25°C. The absorbance of samples was measured at 532 nm through the Tecan Sunrise spectrophotometer (Austria).

### 2.8 Texture profile analysis

The instrumental texture profile (hardness, chewiness, springiness, gumminess, cohesiveness, and resilience) of chicken tender pops was evaluated using a Universal TA‐XT plus texture analyzer (Stable Micro Systems, UK) and its propriety Exponent software, version V.5.1.1.0, as described by de Paiva et al. (2021). A slice of 1 × 1 cm was ligated from the treated samples of chicken tender pops. A double compression cycle test was implemented up to 50% strain compression by means of an aluminum cylinder probe of 3.6 cm diameter at a speed of 1 mm/s to acquire force–time deformation curves. Force versus time plots were used to estimate TPA values that were recorded at 0, 7, 14, and 21 days of storage at room temperature 25°C.

### 2.9 Instrumental color analysis

The instrumental color of chicken tender pops was determined using a Hunter lab calorimeter at 0, 7, 14, and 21 days of refrigerated storage. CIE L* (lightness), a* (redness), and b* (yellowness) of samples were measured (Shahamirian et al., 2019).

### 2.10 Determination of cooking characteristics

Cooking yield and cooking loss expressed the overall cooking characteristics of samples. Samples were air fried for 10 min at 200°C and then cooled at room temperature 25 ± 5°C. The cooking yield and loss were measured as weight differences before and after frying chicken tender pop, following Sunantha and Saroat (2011) and El‐Nashi et al. (2015).

$$\text{Cooking Yield}\;\left(\%\right)=\frac{\text{Weight of Fried Chicken Tender Pops}}{\text{Weight of}\;\mathrm{Raw}\;\text{Chicken Tender Pops}}\times 100$$

$$\text{Cooking Loss}\;\left(\%\right)=\frac{\text{Weight of}\;\mathrm{Raw}\;\text{Sample}-\text{Weight of Fried Sample}}{\text{Weight of}\;\mathrm{Raw}\;\text{Sample}}\times 100$$

### 2.11 Microbiological evaluation

The microbiological analysis for each treatment was performed according to International Standards (APHA, 2001). Maximum recovery solution was prepared by adding 10 g of sample in 90 mL of peptone water in a sterile stomacher bag for 2 min of blending in the stomacher (IUL Instruments, Mod. 1986/470, Spain). After that, serial dilutions up to 10^−3^ were prepared using 1 mL sample in 9 mL of peptone water. The samples were inoculated in particular culture media for enumeration of studied microorganisms: total viable count and psychotropic count (plate count agar; at 37°C for 48 h and 10°C for 5 days, respectively), coliform count (violet red bile agar; at 30°C for 48 h), *Staphylococcus* (Baird–Parker agar with egg yolk and 1% potassium tellurite, Hi‐media, Mumbai, India), *Salmonella* (XLD agar; at 35°C for 24 h), and *Listeria monocytogenes* (Meuller–Hinton agar, HiMedia, Mumbai, India). All microbial counts were calculated as logarithms of colony‐forming units per gram (log cfu/g). *Staphylococcus*, *Salmonella*, and *Listeria* analyses were performed at 0 and 21 days of storage, whereas all other investigations were made for the whole storage period at an interval of 7 days (Honrado et al., 2022).

### 2.12 Sensory evaluation

Sensory evaluation of chicken tender pops was carried out by an untrained consumer panel of 20 individuals using a 9‐point hedonic scale (score of 9 as excellent and 1 as extremely poor) at the Department of Food Sciences, University of the Punjab, Lahore, Pakistan. The items were air fried and served warm for a few minutes before sensory analysis. The samples were evaluated based on their general acceptability, juiciness, flavor, appearance, color, texture, and juiciness. Around 4.30 p.m., sensory evaluation was conducted in a setting with adequate lighting. The panelists offered potable water to rinse their mouths after each sample (Santhi et al., 2020).

### 2.13 Statistical analysis

Acquired data were articulated as the mean values of three replicates, and standard deviations were analyzed statistically by evaluating variance using SPSS version 25.0. Two‐way ANOVA and LSD's post hoc analysis were used for multiple comparisons. For all tests, p‐values of (*p* < .05) were considered statistically significant.

## 3 RESULTS AND DISCUSSIONS

### 3.1 Physiochemical analysis of pomegranate peel powder

The physicochemical composition of PPP is illustrated in Table 2. The percentage of moisture, crude fiber, fat, protein, and ash were 7.28 ± 1.50, 13.91 ± 0.02, 1.61 ± 0.08, 3.78 ± 0.14, and 3.63 ± 0.05, respectively. The results of our studies are in line with those of Kushwaha et al. (2013) and Rowayshed et al. (2013). According to our studies' results, pomegranate peel powder's pH was 4.86 ± 0.02, which was in near accordance with Jalal, Pal, Ahmad, et al. (2018). TPC in the methanolic extract of PPP was 23.59 ± 0.02 mg GAE/g, which is responsible for its excellent radical scavenging properties. Our findings are parallel to those of Jalal, Pal, Hamdani, et al. (2018), who reported the TPC of methanol extract of PPP to be 24.00 mg GAE/g.

**TABLE 2:** Physicochemical chemical composition of PPP.

Moisture	7.28 ± 1.50%
Fat	1.61 ± 0.08%
Protein	3.78 ± 0.14%
Crude fiber	13.91 ± 0.02%
Ash	3.63 ± 0.05%
pH	4.8
TPC	23.59 ± 0.02 mg GAE/g of dry peel

Abbreviation: PPP, Pomegranate Peel Powder.

### 3.2 Physiochemical parameters of chicken tender pops

The incorporation of pomegranate peel powder did not significantly change the pH of chicken tender pop as depicted in Table 3. However, a non‐significant (*p* > .05) decrease was observed in treated samples compared to the control, which could be linked to higher acidity of pomegranate peel powder. During refrigerated storage, pH of all treatments decreased non‐significantly (*p* > .05), possibly due to growth of lactic acid bacteria or the conversion of glycogen into lactic acid. A similar effect was reported by Rupasinghe et al. (2022) for frozen storage of chicken wings marinated with several fruit juices. The water‐holding capacity (WHC) represents the amount of water retained by a product on the application of external force. WHC of treated samples was significantly (*p* < .05) improved by the addition of pomegranate peel powder, which is recorded as 43.60%, 46.36%, 47.87%, and 49.36% for T_0_ (0% PPP), T_1_ (3% PPP), T_2_ (6% PPP), and T_3_ (9% PPP) respectively. Similar results were reported by Akhtar et al. (2015), who described that the incorporation of 3% of PPP in beef sausages enhances the WHC of the products. On the other hand, results indicated a decline in WHC with the passage of storage. However, the decline rate was less in the treated sample, which complies with the results of Rupasinghe et al. (2022), who concluded that WHC of chicken wings marinated with different fruit juices reduced with the progression of storage period.

**TABLE 3:** Physicochemical parameters of chicken tender pops supplemented with different concentrations of pomegranate peel powder during refrigerated storage at 4 ± 2°C for 21 days.

Variables	Storage (days)	Treatments			
		T_0_	T_1_	T_2_	T_3_
pH	0	6.22 ± 0.01^k^	6.22 ± 0.02^j^	6.21 ± 0.02^h^	6.20 ± 0.01^g^
	7	6.22 ± 0.02^m^	6.21 ± 0.02^j^	6.20 ± 0.02^i^	6.19 ± 0.02^g^
	14	6.20 ± 0.02^L^	6.20 ± 0.01^f^	6.20 ± 0.02^e^	6.17 ± 0.03^d^
	21	6.19 ± 0.01^i^	6.20 ± 0.02^c^	6.18 ± 0.01^b^	6.16 ± 0.02^a^
WHC (%)	0	43.60 ± 0.02^f^	46.36 ± 0.03^d^	47.87 ± 0.02^b^	49.36 ± 0.02^a^
	7	41.61 ± 0.01^i^	44.85 ± 0.025^e^	46.36 ± 0.03^d^	47.84 ± 0.02^c^
	14	38.60 ± 0.05^k^	42.36 ± 0.02^h^	44.86 ± 0.01^e^	46.35 ± 0.01^d^
	21	35.12 ± 0.01^L^	39.86 ± 0.02^j^	43.35 ± 0.02^g^	44.84 ± 0.02^e^

*Note*: Values are expressed as mean ± S.D.; means with different letters superscripts are significant (*p* < .05).

Abbreviations: T_0_: Chicken tender pops with PPP; T_1_: Chicken tender pops with 3% PPP; T_2_: Chicken tender pops with 6% PPP; and T_3_: Chicken tender pops with 9% PPP.

### 3.3 Proximate composition of chicken tender pops

The moisture content of chicken tender pops was significantly (*p* < .05) decreased by the addition of pomegranate peel powder ranging from 60.05 ± 0.03% to 55.08 ± 0.005%, attributed to the replacement of meat with dried peel powder. A considerable drop in moisture content was observed during storage associated with evaporation into surroundings, but this decrease was lower in treated samples, as elucidated in Table 4. Sharma and Yadav (2020) also reported reduced moisture content in chicken meat patties prepared with pomegranate peel powder. The fat content of chicken tender pops increased in a non‐significant manner with the progression of the storage period due to the breakdown of lipoprotein into lipids and protein (El‐Nashi et al., 2015). A minor increase in the protein content of treated samples is associated with a low amount of protein in pomegranate peel powder. In contrast, a decrease in protein content with the progression of storage is associated with protein breakdown and removal of water‐soluble amino acids along with moisture removal. Similar results were observed by El‐Nashi et al. (2015) for beef sausages enriched with pomegranate peel powder. The addition of pomegranate peel powder did not affect the ash content of chicken tender pops remarkably; however, a slight increase in ash content was observed for all treatments as the progression of the storage period. The crude fiber content of chicken tender pops considerably increased from 0.256 ± 0.005% to 1.45 ± 0.010%. The enhancement of crude fiber is associated with a higher concentration of dietary fiber in pomegranate by‐products (Rowayshed et al., 2013). Bhol and John Don Bosco (2013) also claimed an increase in the dietary fiber of bread fortified with powder of pomegranate by‐products.

**TABLE 4:** Proximate composition of chicken tender pops supplemented with different concentrations of pomegranate peel powder during refrigerated storage at 4 ± 2°C for 21 days.

Variables	Storage (days)	Treatments			
		T_0_	T_1_	T_2_	T_3_
Moisture (%)	0	60.05 ± 0.03^a^	58.08 ± 0.01^b^	57.04 ± 0.02^c^	55.08 ± 0.01^e^
	7	56.05 ± 0.01^d^	54.04 ± 0.05^f^	53.10 ± 0.01^g^	52.03 ± 0.02^i^
	14	52.07 ± 0.01^h^	50.06 ± 0.01^j^	49.07 ± 0.02^k^	48.11 ± 0.02^L^
	21	48.07 ± 0.02^m^	47.08 ± 0.01^n^	46.09 ± 0.01°	45.08 ± 0.01^p^
Fat (%)	0	16.56 ± 0.02^j^	16.57 ± 0.02^ij^	16.58 ± 0.01^ij^	16.58 ± 0.02^i^
	7	16.77 ± 0.02^f^	16.65 ± 0.01^h^	16.64 ± 0.01^h^	16.64 ± 0.02^h^
	14	17.36 ± 0.01^b^	16.85 ± 0.01^e^	16.74 ± 0.01^g^	16.76 ± 0.02f^g^
	21	17.57 ± 0.02^a^	16.92 ± 0.01^c^	16.88 ± 0.02^d^	16.88 ± 0.01^d^
Protein (%)	0	16.85 ± 0.01^h^	17.74 ± 0.01^c^	17.75 ± 0.01^abc^	17.76 ± 0.02^a^
	7	16.84 ± 0.02^i^	17.73 ± 0.01^f^	17.73 ± 0.02^cd^	17.76 ± 0.01^ab^
	14	16.82 ± 0.01^j^	17.73 ± 0.02^f^	17.72 ± 0.01^de^	17.74 ± 0.02^bc^
	21	16.81 ± 0.02^j^	17.71 ± 0.02^g^	17.70 ± 0.02^e^	17.71 ± 0.01^e^
Ash (%)	0	1.98 ± 0.052^f^	2.37 ± 0.01^e^	2.76 ± 0.02^c^	3.11 ± 0.01^a^
	7	1.98 ± 0.01^f^	2.38 ± 0.02^de^	2.76 ± 0.01^bc^	3.11 ± 0.01^a^
	14	1.98 ± 0.01^f^	2.38 ± 0.01^de^	2.76 ± 0.01^bc^	3.11 ± 0.02^a^
	21	1.20 ± 0.01^g^	2.38 ± 0.02^d^	2.78 ± 0.02^b^	3.12 ± 0.01^a^
Crude fiber (%)	0	0.256 ± 0.01^i^	0.663 ± 0.01^f^	1.04 ± 0.02^d^	1.45 ± 0.01^a^
	7	0.256 ± 0.02^j^	0.662 ± 0.01^g^	1.04 ± 0.01^d^	1.44 ± 0.01^ab^
	14	0.253 ± 0.02^j^	0.660 ± 0.02^g^	1.03 ± 0.01^e^	1.43 ± 0.02^bc^
	21	0.252 ± 0.01^k^	0.657 ± 0.01^h^	1.02 ± 0.02^e^	1.42 ± 0.01^c^

*Note*: Values are expressed as mean ± S.D.; means with different letters superscripts are significant (*p* < .05).

Abbreviations: T_0_: Chicken tender pops with PPP; T_1_: Chicken tender pops with 3% PPP; T_2_: Chicken tender pops with 6% PPP; and T_3_: Chicken tender pops with 9% PPP.

### 3.4 TPC and antioxidant activity (TBARS) of chicken tender pops

**FIGURE 1:**
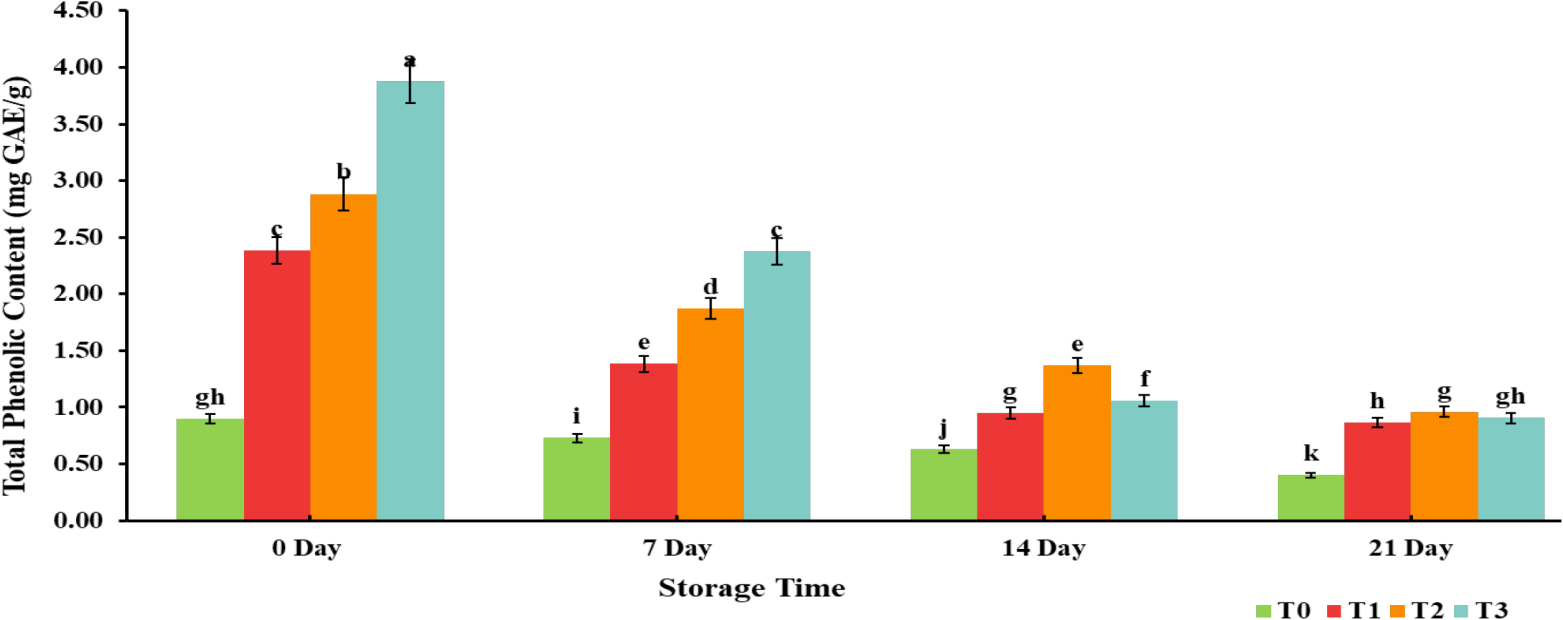
Effect of interaction between treatments and storage days for total phenolic content (mg of GAE/g) of chicken tender pops supplemented with different concentrations of pomegranate peel powder (PPP) during refrigerated storage at 4 ± 2°C for 21 days. Columns labeled with different letters are significantly different, *p* < .05 (*n* = 3). T_0_: control group; T_1_: 3% PPP; T_2_: 6% PPP; and T_3_: 9% PPP.

**FIGURE 2:**
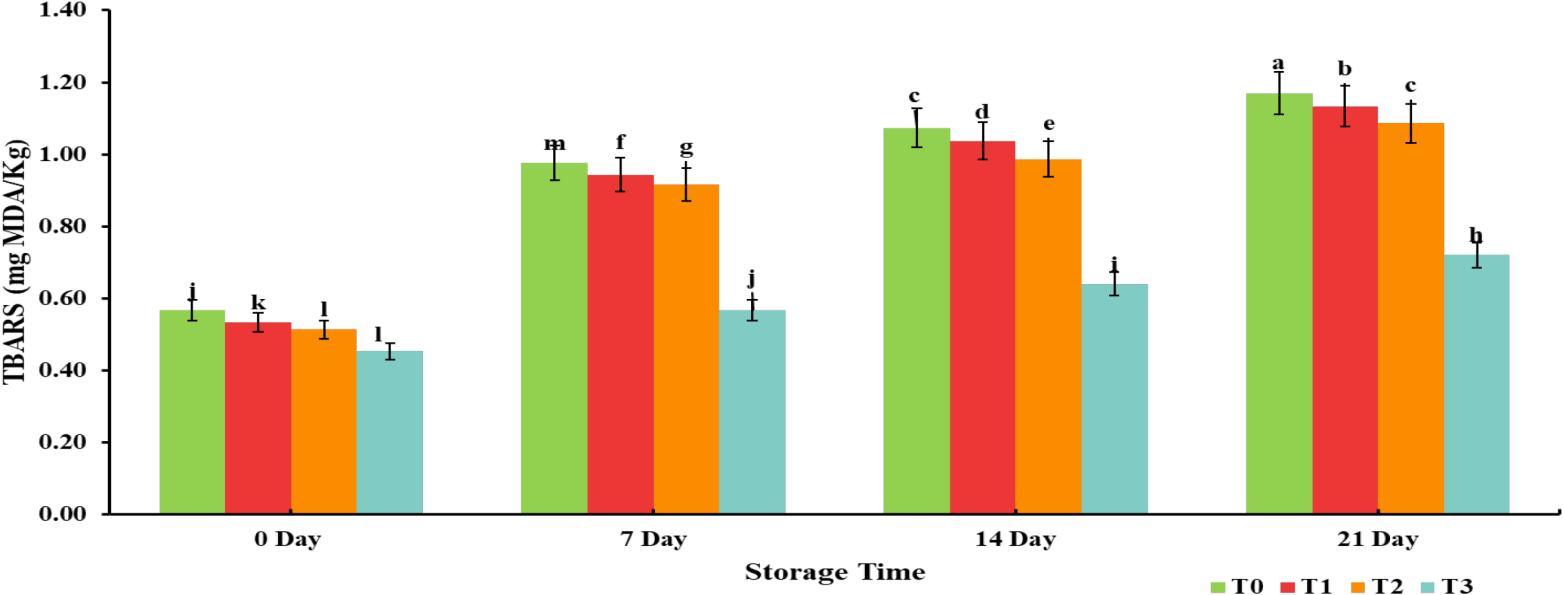
Effect of interaction between treatments and storage days for TBARS (mg of MDA/Kg) of chicken tender pops supplemented with different concentrations of pomegranate peel powder (PPP) during refrigerated storage at 4 ± 2°C for 21 days. Columns labeled with different letters are significantly different, *p* < .05 (*n* = 3). T_0_: control group; T_1_: 3% PPP; T_2_: 6% PPP; and T_3_: 9% PPP.

Antioxidants are compounds that exert a free radical scavenging effect and bind them, preventing degenerative diseases (Villalobos‐Delgado et al., 2019). Pomegranate peel powder is an excellent source of polyphenols that are interlinked to its technological and therapeutic potential (Jalal et al., 2018). Including pomegranate peel powder in chicken tender pops considerably increased the TPC of treated samples ranging from 3.87 mg GAE/g to 0.90 mg GAE/g, as depicted in Figure 1. Devatkal and Naveena (2010) also reported that a higher concentration of phenolic compounds in pomegranate peel contributes to the enhancement of the total phenolic content of the products supplemented with powder. However, with the advancement of the storage period, TPC of chicken tender pops reduced, as reported by Awan et al. (2017), who evaluated the storage stability of garlic‐fortified chicken bites. The presence of little phenolic content (0.90 ± 0.055 mg GAE/g) in the control sample is attributed to spices used for seasoning. TBARS were significantly (*p* < .05) increased for both control and treated samples throughout refrigerated storage. However, the increase in treated samples was significantly lower than the control sample, as presented in Figure 2. Similarly, Vaithiyanathan et al. (2011) witnessed that dipping meat in a phenolic solution significantly (*p* < .05) reduced the TBARS of meat. However, the TBARS increased during the storage period, a consequence of lipid oxidation in muscle foods.

### 3.5 Texture profile analysis of chicken tender pops

The influence of PPP inclusion on the textural parameters of chicken tender pops was scrutinized while refrigerated storage and elucidated in Table 5. A significant (*p* < .05) increase was noticed in hardness, chewiness, and gumminess, whereas cohesiveness and springiness showed a non‐significant (*p* > .05) change in treated samples in relation to control. High concentrations of PPP followed by moisture loss contributed to the increased hardness in treated samples. Conversely, control samples showed low shear force values (Table 5) for chewiness when compared to treated samples (T_1_, T_2_, and T_3_). Our results are consistent with Sharma and Yadav (2020) and Yadav et al. (2016), who manifested a similar effect on the texture profile of processed chicken products by adding several fruits and their by‐products powders. Pietrasik et al. (2020) also reported increased hardness and chewiness in beef burgers supplemented with higher concentrations of pea fibers. In the same way, the gumminess of chicken tender pops was elevated ranging from 28.27 ± 0.05 N to 32.64 ± 0.01 N by the addition of PPP during storage because of increased hardness. Andrés et al. (2017) stated that the inclusion of pomegranate pomace increased the gumminess of lamb patties. However, grape and olive pomace showed considerably higher values than pomegranate pomace. For our studies, springiness and cohesiveness showed a non‐significant variation for control and treated samples, as illustrated in Table 5. López‐Vargas et al. (2014) found similar results for the springiness of beef burgers comprising various ratios of passion fruit powder. In contrast, the literature of Pereira et al. (2022) showed an increase in the springiness of beef burgers added with grape pomace powder. The reduced values of cohesiveness for treated samples may be assigned to the presence of low‐fat content and high crude fiber content in PPP reported by de Alencar et al. (2022). Contrary to our results, de Paiva et al. (2021) reported a significant increase in the cohesiveness of conventional meat nuggets prepared with acerola fruit powder, rosemary, and licorice extract.

**TABLE 5:** Textural profile analysis of chicken tender pops supplemented with different concentrations of pomegranate peel powder during refrigerated storage at 4 ± 2°C for 21 days.

Variables	Storage (days)	Treatments			
		T_0_	T_1_	T_2_	T_3_
Hardness (N)	0	37.57 ± 0.03^m^	47.85 ± 0.02^L^	53.45 ± 0.02^h^	58.40 ± 0.02^d^
	7	37.46 ± 0.02^n^	48.13 ± 0.02^k^	53.73 ± 0.02^g^	58.65 ± 0.02^c^
	14	37.32 ± 0.03°	48.31 ± 0.01^j^	53.91 ± 0.03^f^	58.84 ± 0.02^b^
	21	36.30 ± 0.03^p^	48.49 ± 0.02^i^	54.16 ± 0.03^e^	59.03 ± 0.03^a^
Chewiness (N)	0	17.56 ± 0.02^p^	24.69 ± 0.03^L^	25.63 ± 0.02^h^	26.61 ± 0.02^d^
	7	17.61 ± 0.02°	24.78 ± 0.02^k^	25.72 ± 0.01^g^	26.71 ± 0.01^c^
	14	17.66 ± 0.03^n^	24.87 ± 0.03^j^	25.81 ± 0.02^f^	26.80 ± 0.01^b^
	21	17.71 ± 0.03^m^	24.96 ± 0.02^i^	25.91 ± 0.02^e^	26.89 ± 0.03^a^
Gumminess (N)	0	21.57 ± 0.01^p^	28.27 ± 0.02^L^	30.24 ± 0.02^h^	32.25 ± 0.01^d^
	7	21.65 ± 0.01°	28.40 ± 0.01^k^	30.37 ± 0.01^g^	32.38 ± 0.01^c^
	14	21.73 ± 0.01^n^	28.53 ± 0.02^j^	30.50 ± 0.02^f^	32.51 ± 0.02^b^
	21	21.81 ± 0.02^m^	28.66 ± 0.02^i^	30.63 ± 0.0^1e^	32.64 ± 0.01^a^
Springiness	0	0.87 ± 0.02^c^	0.85 ± 0.03^cd^	0.83 ± 0.02^de^	0.80 ± 0.02^e^
	7	0.88 ± 0.03^b^	0.87 ± 0.04^c^	0.85 ± 0.04^cd^	0.82 ± 0.03^de^
	14	0.88 ± 0.03^b^	0.89 ± 0.02^ab^	0.87 ± 0.03^c^	0.84 ± 0.03^d^
	21	0.91 ± 0.02^a^	0.91 ± 0.03^a^	0.89 ± 0.02^ab^	0.86 ± 0.04^cd^
Cohesiveness (%)	0	0.52 ± 0.03^a^	0.50 ± 0.02^ab^	0.48 ± 0.01^bc^	0.46 ± 0.02^c^
	7	0.51 ± 0.02^ab^	0.49 ± 0.01^b^	0.47 ± 0.02^bc^	0.45 ± 0.03^cd^
	14	0.51 ± 0.02^ab^	0.47 ± 0.02^bc^	0.46 ± 0.01^c^	0.45 ± 0.03^cd^
	21	0.49 ± 0.03^b^	0.47 ± 0.01^bc^	0.46 ± 0.02^c^	0.43 ± 0.02^d^

*Note*: Values are expressed as mean ± S.D.; means with different letters superscripts are significant (*p* < .05).

Abbreviations: T_0_: Chicken tender pops with PPP; T_1_: Chicken tender pops with 3% PPP; T_2_: Chicken tender pops with 6% PPP; and T_3_: Chicken tender pops with 9% PPP.

### 3.6 Instrumental color analysis of chicken tender pops

**FIGURE 3:**
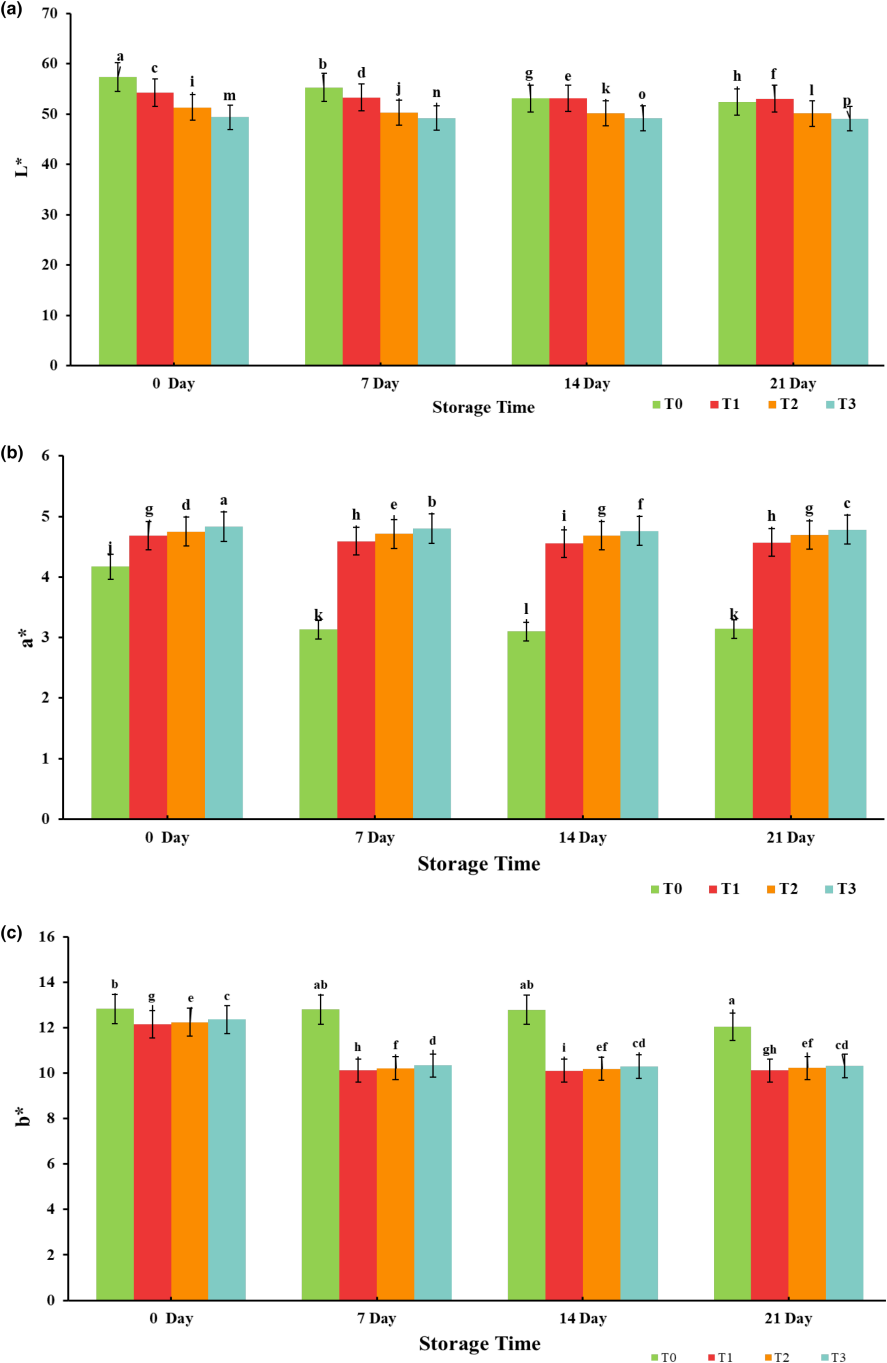
Effect of interaction between treatments and storage days for instrumental color (L*, a*, and b*) a, b, and c, respectively, of chicken tender pops supplemented with different concentrations of pomegranate peel powder (PPP) during refrigerated storage at 4 ± 2°C for 21 days. Columns labeled with different letters are significantly different, *p* < .05 (*n* = 3). T_0_: control group; T_1_: 3% PPP; T_2_: 6% PPP; and T_3_: 9% PPP.

Product color is regarded as the primary attribute that directly affects the consumer's purchasing intention and makes the product more eye appealing. The incorporation of PPP significantly (*p* < .05) affected the color of chicken tender pops during storage. A significant decrease was observed in lightness (L*) of treated samples as compared to control (as shown in Figure 3a); for the whole storage period, reduction in L* can be linked to the darker color of PPP and pigment dilution that leads to darker product color. Sáyago‐Ayerdi et al. (2009) reported a lower value of L* for chicken burgers enriched with grape dietary fibers. Similarly, the literature of Shahamirian et al. (2019) showed inhibition of brightness by the addition of pomegranate juice and rind powder extract in frozen burgers in relation to control. All treatments presented a drop in redness (a*), but it was more significant in the control sample than in treated samples (as illustrated in Figure 3b). The decline in a* is interlinked to the oxidation of myoglobin followed by accumulation of metmyoglobin that imparts a darker‐brown color to meat products. Similar results were observed by Devatkal et al. (2010), who added pomegranate seed extract to beef patties. However, more intense color was evident in control (3.14) than in treated samples 4.57, 4.69, and 4.78 at the end of storage, which can be attributed to the antioxidant potential of PPP. The addition of olive and grape pomace extracts to meat patties retained the intensity of red color in contrast to control sample (Andrés et al., 2017). The inclusion of PPP significantly (*p* < .05) reduced the yellowness (b*) in treated samples 12.15–12.37 than control 12.83. However, heterogeneous variation was observed for b* throughout the storage as depicted in Figure 3c. A significant drop was noticed for the treated sample on day 7, whereas on day 21 non‐significant (*p* > .05) increase was observed. Pereira et al. (2022) studied beef burgers' quality and sensory attributes, and correspondingly noticed a decrease in b* values as the proportion of grape pomace meal augmented. Firuzi et al. (2019) also reported a reduction in b* of frankfurter supplemented with various pomegranate fruit extracts. Contrarily, according to Estévez et al. (2005), increased b* was seen in frankfurter samples containing rosemary essential oil during chilled storage.

### 3.7 Cooking characteristics of chicken tender pops

Cooking yield and cooking loss of tender chicken pop prepared with 0%, 3%, 6%, and 9% pomegranate peel powder were assessed, and the results are elucidated in Table 6. There was a considerable increase in the cooking yield of the treated sample compared to the control sample. The upsurge in cooking yield is attributed to the water‐retaining properties of pomegranate peel powder. Analogous effects were reported by Mashau et al. (2021) for the cooking yield of ground beef supplemented with moringa leaves powder. On the other hand, adding pomegranate peel powder reduced the cooking loss in developed products from 17.17% to 12.68%. According to El‐Nashi et al. (2015), the reduction in cooking loss is attributed to the WHC of pomegranate peel powder. However, the advancement of storage resulted in a significant decrease in cooking yield and a significant increase in cooking loss.

**TABLE 6:** Cooking characteristics of chicken tender pops supplemented with different concentrations of pomegranate peel powder during refrigerated storage at 4 ± 2°C for 21 days.

Variables	Storage (days)	Treatments			
		T_0_	T_1_	T_2_	T_3_
Cooking yield (%)	0	82.58 ± 0.12^ef^	84.67 ± 0.05^d^	86.48 ± 0.15^b^	88.40 ± 0.04^a^
	7	78.52 ± 0.051^j^	83.19 ± 0.03^e^	84.43 ± 0.03^d^	85.43 ± 0.02^c^
	14	74.52 ± 0.02^k^	81.64 ± 0.04^g^	82.93 ± 0.02^ef^	82.36 ± 0.03f^g^
	21	71.51 ± 0.02^L^	78.64 ± 1.70^j^	80.83 ± 0.02^h^	79.85 ± 0.02^i^
Cooking loss (%)	0	17.17 ± 0.05^L^	15.30 ± 0.05^n^	14.88 ± 0.05°	12.68 ± 0.50^p^
	7	20.64 ± 0.02^g^	17.77 ± 0.02^j^	17.35 ± 0.02^k^	16.57 ± 0.03^m^
	14	23.56 ± 0.03^c^	20.75 ± 0.03^f^	19.84 ± 0.03^h^	19.57 ± 0.02^i^
	21	28.46 ± 0.02^a^	24.24 ± 0.02^b^	23.32 ± 0.03^d^	23.03 ± 0.02^e^

*Note*: Values are expressed as mean ± S.D.; means with different letters superscripts are significant (*p* < .05).

Abbreviations: T_0_: Chicken tender pops with PPP; T_1_: Chicken tender pops with 3% PPP; T_2_: Chicken tender pops with 6% PPP; and T_3_: Chicken tender pops with 9% PPP.

### 3.8 Microbiological studies of chicken tender pops

**FIGURE 4:**
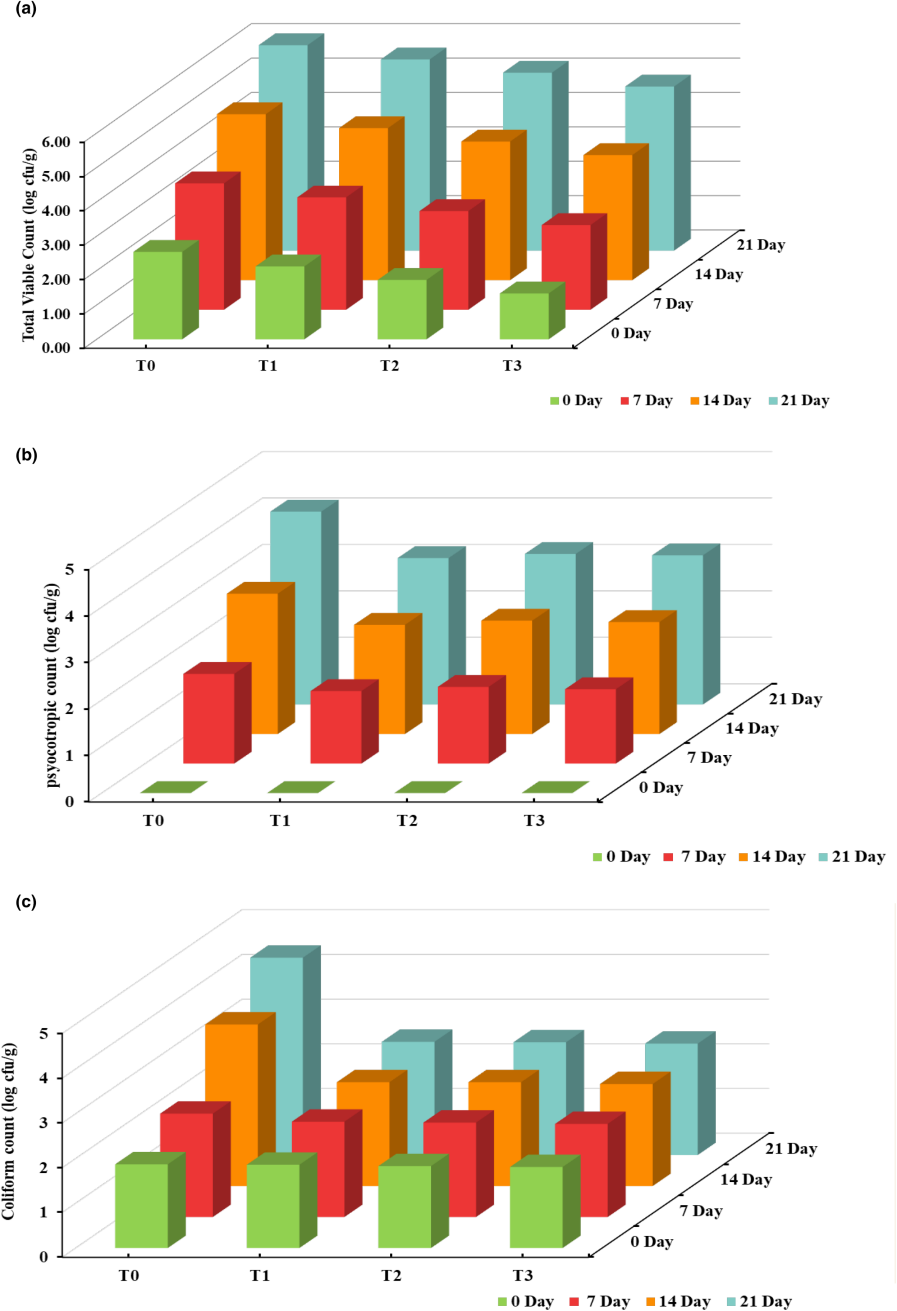
Effect of interaction between treatments and storage days for (a) total viable count (log cfu/g), (b) psychotropic count (log cfu/g); and c: coliform count (log cfu/g) of chicken tender pops supplemented with different concentrations of pomegranate peel powder (PPP) during refrigerated storage at 4 ± 2°C for 21 days. T_0_: control group; T_1_: 3% PPP; T_2_: 6% PPP; and T_3_: 9% PPP.

The effect of PPP supplementation on the microbiological safety of chicken tender pops during storage was investigated through the assessment of total viable count, psychotropic count, and coliform count along with *Staphylococcus*, *Salmonella*, and *Listeria monocytogenes*. The total viable count (TVC) for chicken tender pops significantly (*p* < .05) increased with the advancement of the storage period in all treatments as shown in Figure 4a. However, the increase in the TVC of treated samples was slightly less than that of the control sample. The TVC observed for the control sample was 2.54 ± 0.02 log cfu/g, whereas TVC for the treated sample (T3) was recorded as 1.33 ± 0.03 cfu/g. A similar trend was observed for the psychotropic count of chicken tender pops shown in Figure 4b, illustrating a significantly lower increase in the treated sample. The inhibitory effect is ascribed to the presence of different phenolic compounds in pomegranate peel that exerts an antimicrobial effect on the product. The results agree with Sharma and Yadav (2020), who observed the same inclination of microbial count for chicken patties supplemented with numerous by‐products of the pomegranate fruit. Chandralekha et al. (2012) also observed a significant decline in the microbial count of meatballs prepared by incorporating 5% pomegranate rind powder stored under refrigerated conditions. The coliform count decreased with the addition of PPP, as depicted in Figure 4c, which supports the potential of PPP as a natural preservative, although there was an increase in the number of coliforms with the progression of storage interval. The pragmatic results are in accordance with Kanatt et al. (2010) and Al‐Zoreky (2009), who reported that the antimicrobial action of PPP against different bacterial species is due to its interference with bacterial protein synthesis.

On the other hand, *Staphylococcus*, *Salmonella*, and *Listeria* were investigated as human pathogens at the beginning and the end of the study. The results showed negative values for all of these bacterial pathogens during the entire storage period hence conforming to safety legislation, Regulation EC No 2073/2005 (European Food Safety Authority, 2010). Furthermore, experimental results are supported by Honrado et al. (2022) and El‐Nashi et al. (2015), who witnessed similar results for low‐fat rabbit sausages and beef sausages developed using various concentrations of PPP.

### 3.9 Sensory evaluation

**FIGURE 5:**
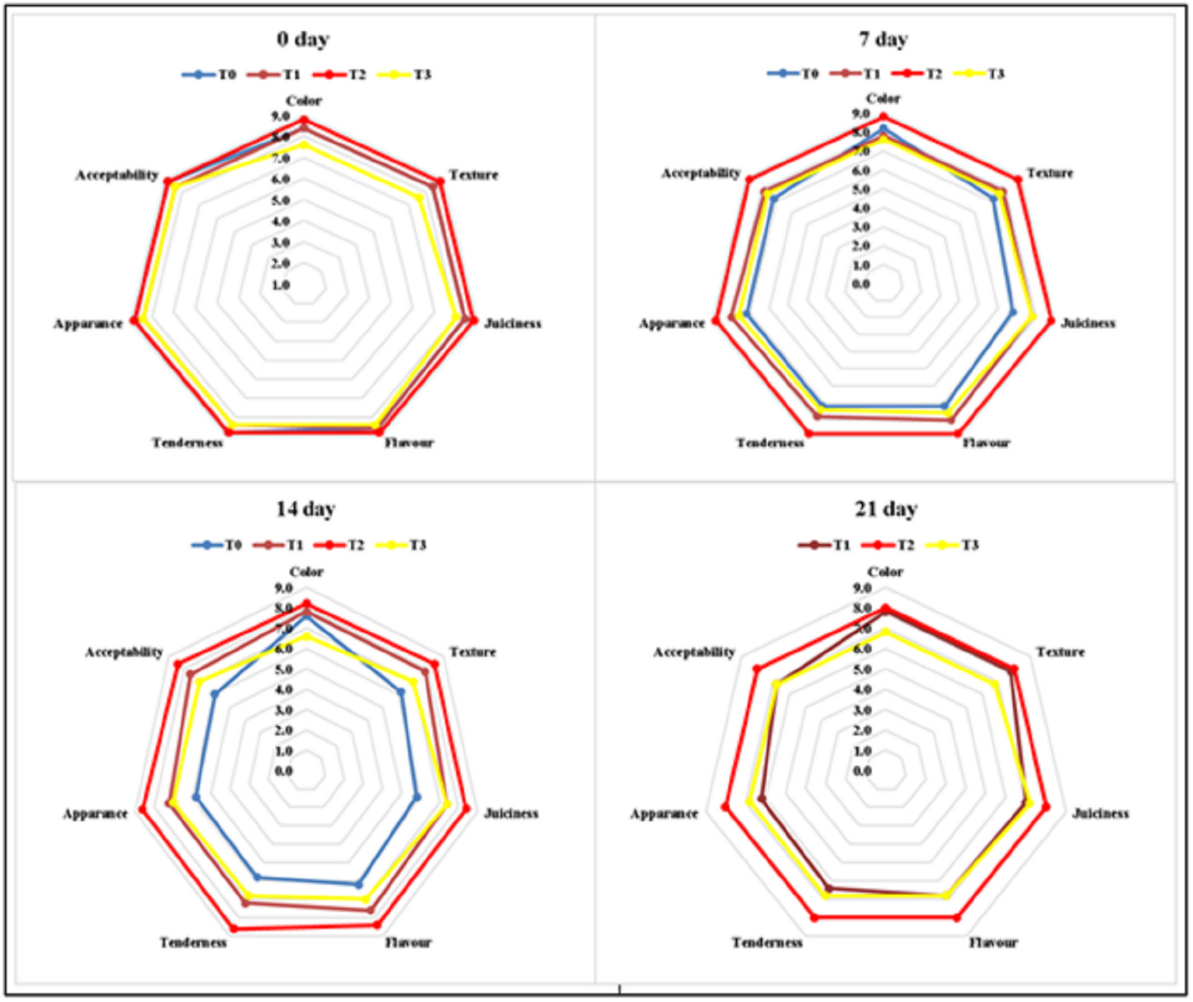
Effect of interaction between treatments and storage days for sensory scores of texture, juiciness, flavor, tenderness, appearance, and overall acceptability of chicken tender pops supplemented with different concentrations of pomegranate peel powder (PPP) during refrigerated storage at 4 ± 2°C for 21 days. T_0_: control group; T_1_: 3% PPP; T_2_: 6% PPP; and T_3_: 9% PPP.

Sensory attributes (appearance, color, texture, flavor, juiciness, tenderness, and overall acceptability) of prepared treatments (T_0_, T_1_, T_2_, and T_3_) containing different concentrations of pomegranate peel powder at different storage intervals are represented in Figure 5. Results indicated that the inclusion of pomegranate peel powder significantly improved the product's sensory attributes during storage with increased overall acceptability. However, in T_3_ (9% PPP), darker red color was observed with a slight dryness with the progression of storage. In general, the best sensory scores were received by T_2_ chicken tender pops containing 6% PPP. Hence, the panelists declared chicken tender pops with 6% pomegranate peel powder (T_2_) as the best treatment for all organoleptic properties during the study period. The results for sensory evaluation of color and textural properties (juiciness and tenderness) can be correlated with instrumental color analysis and texture profile analysis. Sensory scores for color were decreased as an increase in PPP concentration and results are analogous to L* and a* values which show increased darkness in product due to myoglobin oxidation and PPP color. Furthermore, instrumental texture analysis showed increased hardness and dryness because of moisture loss that will ultimately lower the juiciness and tenderness of the product.

### 3.10 Heatmap and hierarchical analysis

**FIGURE 6:**
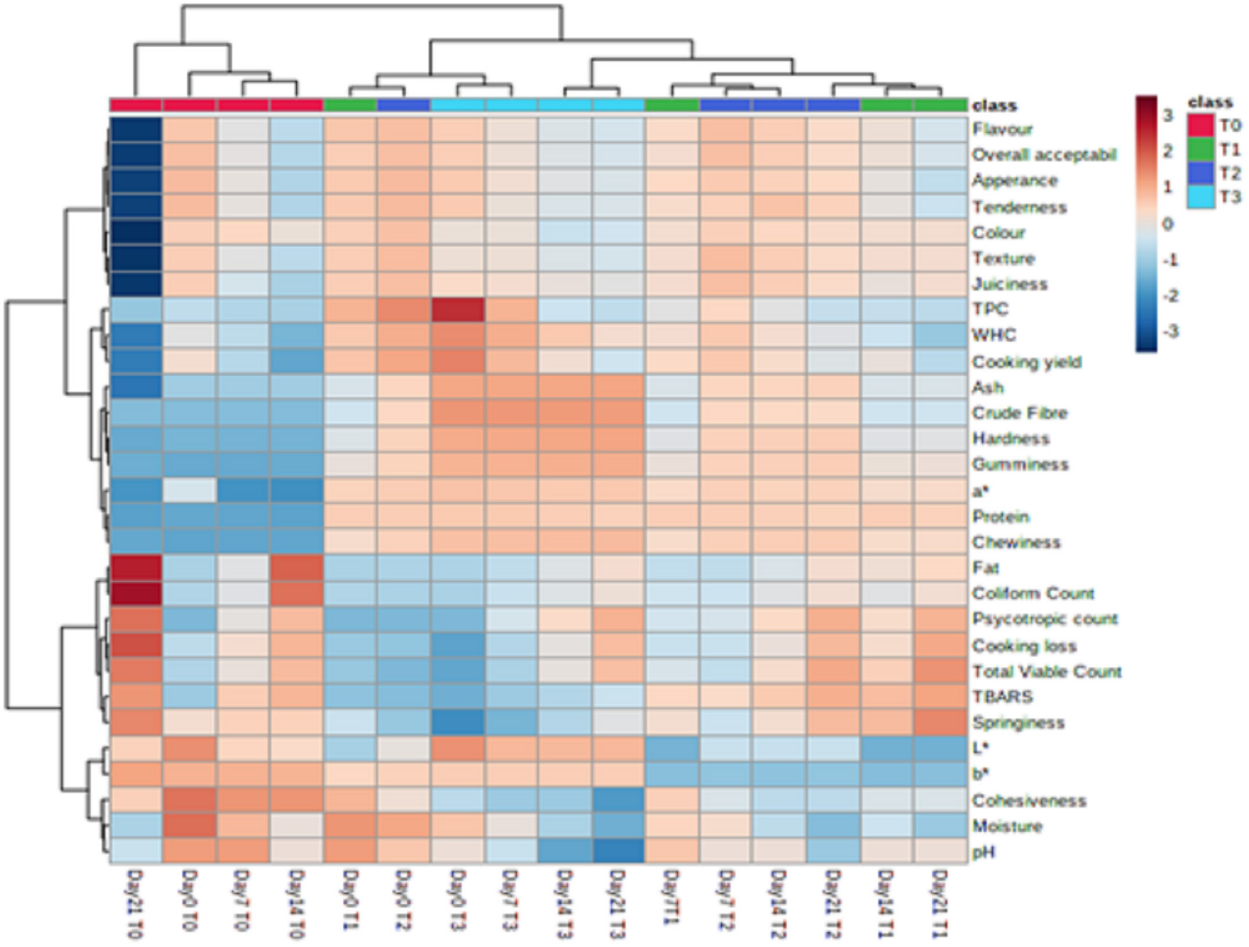
Heatmap analysis illustrating the correlation of all parameters in chicken tender pops supplemented with pomegranate peel powder during 21 days of storage.

A heatmap was generated to analyze all variables pertaining to storage time and pomegranate peel powder concentration (Figure 6). In addition to data classification, a heatmap entails color comparison to make the findings more intrusive. Multiple separate clusters were observed for key variables, illustrating variances across them. The darkest red color, showing the highest concentration, was revealed for TPC in T_3_ (9% PPP), accompanied by coliform count and fat in the control sample at day 0. A slightly lighter color was depicted by T_2_ (6% PPP) and T_3_ at Day 0 for TPC and WHC and cooking yield, respectively.

Similarly, T0 indicated slight variation for coliform and fat on day 14, whereas T0 showed cooking loss on day 21. The moderate‐color cluster was noted in T_3_ for ash, crude fiber, gumminess, and hardness for the whole storage period. On the other hand, neutral color was displayed for moisture in T_3_ and T_0_ on days 7 and 14, respectively. Likewise, neutral color was noticed for organoleptic properties (flavor, tenderness, appearance, and overall acceptability) in T_0_ on day 7. The minimum values in a cluster were indicated by the dark blue color that was spotted in the control sample for all sensory attributes (appearance, color, flavor, tenderness, juiciness, texture, and overall acceptability) on the 21st day of storage.

### 3.11 Correlation and principal component analysis (PCA)

**FIGURE 7:**
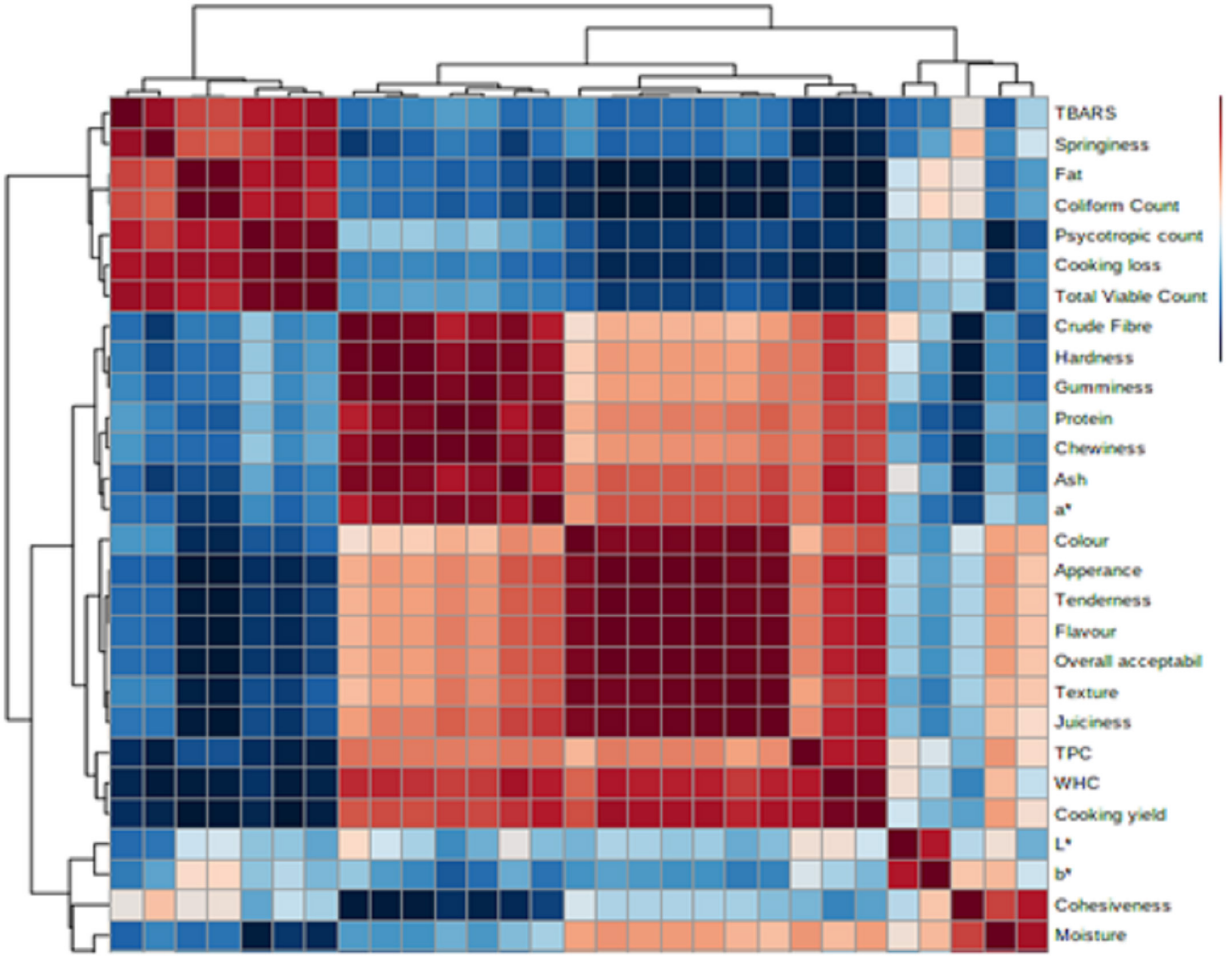
Correlation among various parameters of chicken tender pop samples supplemented with pomegranate peel powder during 21 days of storage.

A regression analysis was performed to evaluate the correlation among the results of conducted assays on chicken tender pop samples supplemented with pomegranate peel powder shown in Figure 7. A significant positive correlation was observed between coliform count and fat. Similarly, the parameters hardness, chewiness, and gumminess were also observed to be positively correlated. Correlation analysis revealed that all the sensorial attributes (color, appearance, tenderness, flavor, texture, juiciness, and overall acceptability) were significantly correlated with each other in a positive correlation. Water‐holding capacity and cooking loss were also observed to be positively correlated. A comparatively neutral correlation was found between pH and organoleptic characters. Sensory properties were observed to be negatively correlated with coliform count and psychotropic count. Crude fiber, hardness, and gumminess depicted a negative correlation with cohesiveness indicating an inverse relation. Total plate count and cooking yield were also observed to be in a negative correlation with cooking yield.

**FIGURE 8:**
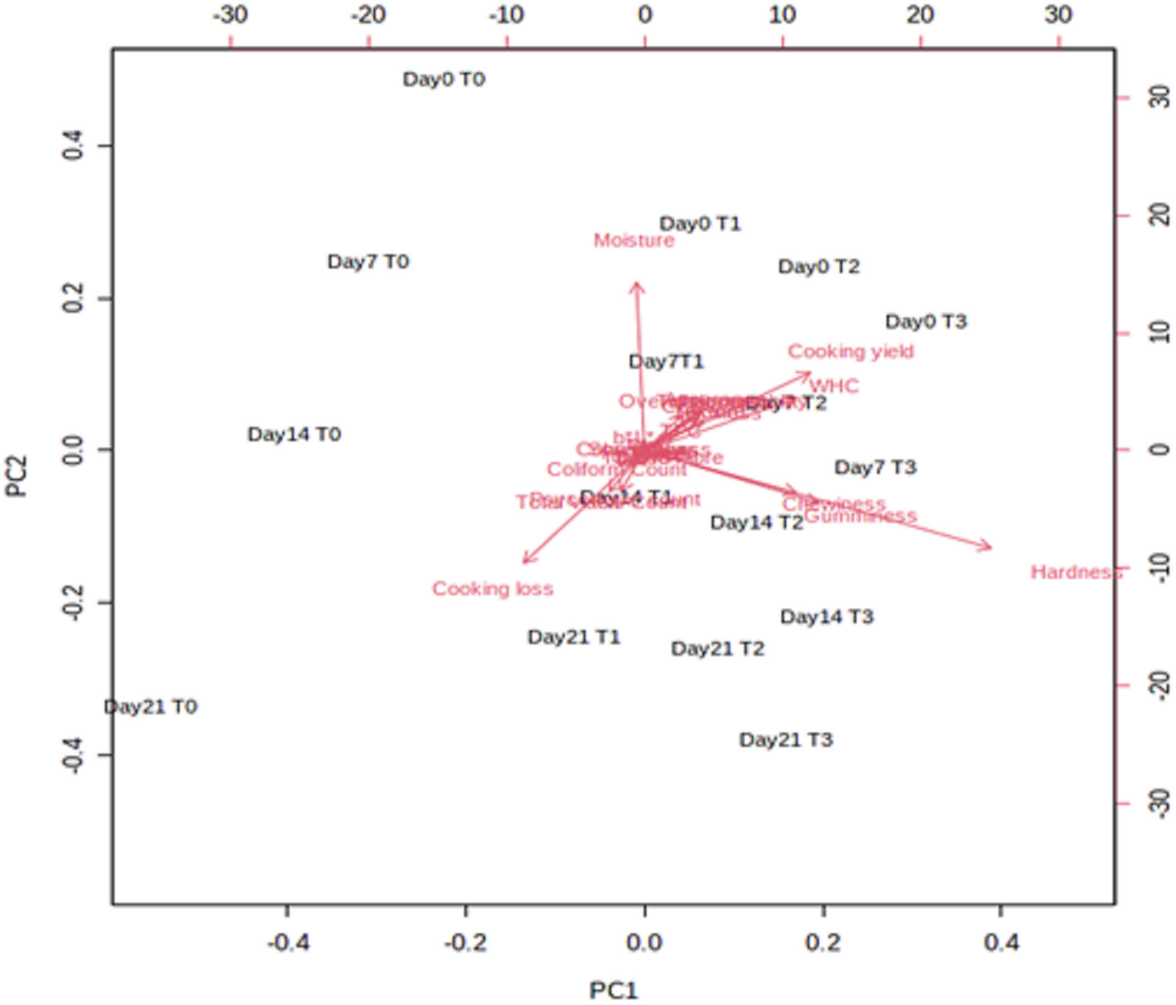
Principal component analysis (PCA) of different parameters of chicken tender pops supplemented with pomegranate peel powder during 21 days of storage.

Principal component analysis (PCA) was characterized by reducing a large number of variables to a small number of comprehensive variables, accurately expressing the total amount of data. Signal intensities are used in PCA to highlight the differences between the parameters that were taken into consideration. Figure 8 presents the results of the principal component analysis (PCA) of the pomegranate peel powder–enriched chicken tender pop samples. PCA illustrated maximum quantities of coliform count, b* (yellowness/blueness) value, and cooking loss of sample T3 (6% PPP) at day 7. Cooking yield and WHC revealed comparatively lower values for T1 on day 7. A similar trend was observed for chewiness and gumminess of T2 samples at day 14. Cooking loss and hardness were further reduced for samples T1, T2, and T3 at day 21. Minimum quantities were observed for control sample at day 0.

## 4 CONCLUSION

Incorporating pomegranate peel powder reduced the moisture content and significantly improved the product's water‐holding capacity, ultimately enhancing the product's sensory attributes. Furthermore, the inclusion of PPP enhanced crude fiber content in treated samples compared to the control group. Significant elevation in total phenolic content indicated the potential of PPP to be used as a natural antioxidant. In contrast, reduced TBARS elucidated the positive impact of PPP on lipid auto‐oxidation. Hardness, chewiness, and gumminess were considerably affected, whereas springiness and cohesiveness showed minor variations. In addition, PPP retarded pigment oxidation was indicated by retained red color during storage. The reduced microbial load validates the antimicrobial potential of PPP ascribed to the presence of polyphenols and flavonoids. Pomegranate peel powder significantly improves the sensory attributes, but up to a specific limit, as for our study, it was at 6%. Based on our study, it can be concluded that chicken products supplemented with PPP have improved nutritional and sensory profiles and can be naturally preserved for up to 3 weeks at refrigeration temperature.

## AUTHOR CONTRIBUTIONS

**Zunaira Basharat:** Conceptualization (equal); formal analysis (equal); investigation (equal); methodology (equal). **Maryam Imran:** Investigation (equal); visualization (equal); writing – review and editing (lead). **Naeem Fatima:** Investigation (supporting); visualization (supporting); writing – review and editing (supporting). **Muhammad Wasim Sajid:** Conceptualization (equal); formal analysis (equal); investigation (equal); methodology (equal); writing – original draft (equal). **Muhammad Rizwan Tariq:** Conceptualization (equal); formal analysis (equal); methodology (equal); validation (equal). **Shinawar Waseem Ali:** Conceptualization (equal); data curation (equal); investigation (equal); writing – review and editing (equal). **Zujaja Umer:** Formal analysis (equal); investigation (equal); methodology (equal); writing – review and editing (equal). **Humphrey Garti:** Conceptualization (equal); data curation (equal); formal analysis (equal); methodology (equal); writing – review and editing (equal).

## FUNDING INFORMATION

We did not receive any funding for this work.

## CONFLICT OF INTEREST STATEMENT

The authors have no competing interest to declare.

## Data Availability

The data that support the findings of this study are available from the corresponding author upon reasonable request.
